# Regulating Gamete Donation in the U.S.: Ethical, Legal and Social Implications

**DOI:** 10.3390/laws4030352

**Published:** 2015-07-28

**Authors:** Maya Sabatello

**Affiliations:** Center for Research on Ethical, Legal and Social Implications of Psychiatric, Neurologic & Behavioral Genetics, Department of Psychiatry, Columbia University, 1051 Riverside Drive, New York, NY 10032, USA; Tel.: +1-646-774-8632

**Keywords:** gamete donation, right to know, privatized market, relativism, medical ethics

## Abstract

This article explores the practice of gamete donation in the U.S. having in mind the larger question of what do we as a society owe children born as a result (donor-conceived children). Do recipient-parents have a duty to tell their donor-conceived child about his/her genetic origins? Should the identity of the donor be disclosed or remain anonymous? Does the child have a *right* to know her conception story and to receive information, including identifying information, about the donor? Furthermore, if a donor-conceived child has a right to know, who has the duty to tell her/him about it? The Article underscores the ethical, legal and social dilemmas that arise, comparing and contrasting with international developments in this arena. It highlights the market-based and more specific medical justifications for regulating this field, explores the emerging so-called right of the child to know his/her genetic origins (“the right to know”), and considers the challenges such a right evokes to existing legal culture and principles of medical ethics in the U.S. as well as other broader societal implications of such a right.

## 1. Introduction

The practice of gamete donation has received growing attention in the past two decades. As an increasing number of individuals and couples resort and have resorted to gamete donation [1,2], and the number of donor-conceived children born as a result are reaching maturity, many are grappling with the question as to how to address the dilemmas that arise [3–8]. Do recipient-parents have a duty to tell their donor-conceived child about his/her genetic origins? Should the identity of the donor be disclosed or remain anonymous? Does the child have a *right* to know her conception story and to receive information, including identifying information, about the donor? Furthermore, if a donor-conceived child has a right to know, who has the duty to tell her about it?

In response to such questions, most legislatures in the Western world have taken steps to regulate the field [9–16]. Beyond informed consent of gamete donors and recipient parents, they have set limitations on the potential donors, the number of recipient families, the price for gametes, and the recording of the donor's information [17]. An increasing number of countries have further reversed the long-held policy of gamete donors' anonymity, collecting gamete donors' identifying information and requiring their consent to be contacted in the future by any resulting donor-conceived child (see Section 3).

In contrast, the market for assisted reproductive technologies in the U.S., including gamete donation, is barely regulated. There is no comprehensive law relating to assisted reproductive technologies, and the U.S. Food and Drug Administration (FDA) included gamete donations under its eligibility regulations for Donors of Human Cells, Tissues, and Cellular and Tissue-Based Products (2007) [18]. These regulations require a review of “relevant medical records” and impose screening of donated gametes for predominantly communicable and infectious diseases (e.g., HIV, Hepatitis C), including six months quarantine and retesting before use of anonymous donations. Similarly, the American Society of Reproductive Medicine can only recommend that donors' information is recorded and preserved. The regulation of private fertility clinics and gamete banks by individual states is also often lacking, and among those that have crafted regulations—there is great variation as to the collection, preservation, and release of donors' information [5,19]. Given this stark difference between the U.S. and other developed, Western countries and their regulations of gamete donation, it is important to consider the justifications for this emerging international trend and its relevance to the U.S., as well as what implications such a change would entail for current principles of medical ethics.

This paper aims to explore this issue, having in mind the larger question of what we, as a society, owe children born as a result of ART, especially from gamete donation. It endeavors to underscore the arising ethical, legal, and social dilemmas, rather than provide a comprehensive legal framework, which is beyond the scope of this article. Section 2 considers the context of gamete donation in the U.S. and highlights the medical justifications for regulating the field. Whether these justifications are—or should be—further translated into the emerging so-called right of the child to know his/her genetic origins (“the right to know”) is explored in Section 3. In addition, I present three main challenges such a right presents to legal framework and existing principles of medical ethics in the U.S. In Section 4, I consider two broader implications of the right to know. Finally, in Section 5, I make a few recommendations and draw some conclusions.

## 2. Gamete Donation in Context: The U.S

Current calls to revise—or to more strictly regulate—the current market in the U.S. have to be considered in light of the tremendous medical, societal, and legal shifts that have occurred in the past few decades with regard to gamete donation. These include the number of parents who resort to gamete donation, and more importantly, the millions of children (worldwide) that have been born as a result [1]; the increased societal acceptance of the procedure, which paved the way to its legalization, especially the regulation of the status of donor-conceived children and the recipient parents as a family unit [20–23]; and the development of medical guidelines that not only recognize infertility as a medical condition that can (and in many national healthcare systems, when requested, also should) be treated, but also that require service providers to follow certain standards such as transparent recruitment of donors [7], screening of gametes for various medical (especially infectious) diseases, and the securing of donors' and recipient parents' informed consent [18].

Of course, not all countries allow gamete donation, and the exact procedures for carrying out the practice vary significantly across countries [24–26]. Indeed, as in other contexts of assisted reproductive technologies (e.g., surrogacy), laws and policies about gamete donation reflect many moral, legal, and cultural facets and countries strike a different balance between donors and recipient parents' privacy interests and reproductive freedom and the child's rights and best interests. However, even among those countries that allow gamete donation (of at least sperm), the U.S. market is unique. First, gamete donation in the U.S., as all other assisted reproductive technologies, is an outright, and undoubtedly thriving, commercial activity. It generates billions of dollars per year [27], donors' recruitment is often made in public spaces (e.g., campuses, and increasingly, online), and uniquely to the U.S., donors, especially egg donors, are well compensated for their gametes [28,29] ^1^. In the absence of a centralized system to record gamete donations, donors may also donate multiple times and in various clinics, thus further increasing their profit from the donation. Second, and related, the “Wild Wild West” in the U.S. [30] ^2^ serves as a fertile ground for the creation of many thousands of (traditional and nontraditional [1]) families that would not have otherwise existed. In addition to American individuals and couples (both heterosexual and same-sex) who access assisted reproductive technologies, including gamete donation, the U.S. serves as a leading actor in the international reproductive tourism market [28]. Indeed, the estimate of donor-conceived children who are born in the U.S. each year is 30,000–60,000 children from sperm donation [1] and over 8,000 from egg donation [31]. Notwithstanding the moral condemnation some may raise for this business, this approach fits the U.S. creeds. As I elaborate in Section 3, the U.S. does not endorse the formal recognition of children as subjects with rights as other Western nations and prioritizes the rights and interests of adults, especially parents, over those of children. As a society that is highly invested in (adults') freedoms—reproductive freedom, privacy rights, and free market are especially relevant—many in the U.S. believe that the present system is satisfactory.

Nonetheless, I suggest that these arguments can stand only as long as the interests of donor-conceived children are neglected. Certainly, it is highly controversial to talk about donor-conceived children as patients at the time of gamete donation or conception and even after implantation in the womb. The U.S. has long been split on the moral and legal status of such entities, as can be discerned from the ongoing debate about abortion. However, donor-conceived children are clearly the end goal of the process, and implications related to their circumstances of conception are likely to emerge after birth, when the child certainly has moral and legal status in society. Consideration of the interests of donor-conceived children is thus crucial for an informed discussion.

In particular, an interest in accessing information about the donor may be especially relevant when donor-conceived children show medical symptoms, which may be the result of a genetic condition that the child inherited from the donor [3,4]. Although the genetic cause is not yet confirmed at this point, retrieving the donor's information in such instances would arguably allow for quicker diagnosis, treatment, and adoption of preventative measures [4]. The sense of medical urgency arises also with asymptomatic children. In this regard, donors' information may enable taking preventative measures, if needed (and possible), avoiding (future) consanguineous relationships between donor-conceived siblings, which increase the risk of genetic disorders if the siblings procreate [32,33], and allowing recipient parents and the donor-conceived child to make more informed reproductive choices [4].

To be sure, these arguments are not as absolute as they may initially appear. Many genetic disorders are multi-genetic and multi-factorial; they involve complex groups of genes not yet identified, as well as yet-unknown gene-environment interactions and epigenetic processes that are extremely difficult to decode [34]. Other genetic disorders are *de-novo*: they present for the first time as a result of a mutation in a germ cell of the fertilized egg itself, and cannot be attributed to the genetic parents [19]. Moreover, advances in genetic testing, especially genomic testing (such as whole genome sequencing and whole exome sequencing), are likely (or hoped) to make family history more redundant. These technologies may confirm a diagnosis that is based on known genetic disorders, provide information of individuals' proclivity for such genetic conditions, and reveal information about genetic relatedness and carrier status without the knowledge of a family history. In the U.S., these technologies may further be more accessible to recipient parents and donor-conceived children than elsewhere because of the thriving market of direct-to-consumer genetic testing companies, which may test for ancestry and increasingly, for health conditions [35]. As a result of this market, not only have the costs of genetic testing have plummeted, but given that these companies rarely posit limitations on the age of their consumers [36], recipient parents and donor-conceived children may use their services. While Europeans may not view this market favorably [37], American primary care providers and the public are increasingly aware and supportive of these services [38]. Finally, and importantly, as Robert Klitzman points out: genetic diagnosis also does not mean that an effective treatment is available (so-called “diagnostic misconception”) [39]. Thus, regardless of whether genetic testing is provided in medical or commercial settings, the practical usefulness of having a genetic diagnosis may be questioned as well.

Nonetheless, the rationales for regulating the market of gamete donation in the U.S. are grounded in both general and more specific deficits that arise in this market. In general, regulations of economic activities in the U.S. take place predominantly to protect weak consumers from strong service providers, including through addressing the problem of asymmetric information that these actors have [40]. In the market for gamete donation, the imbalance of information is clear: donors and clinics may hold significantly more information than the recipient parents, who are ultimately forced to accept the lack of information if they want to proceed with the gamete donation. The considerable vulnerability of prospective parents in accessing reproductive technologies, which beyond information includes financial and psychological costs [1,41], should not be ignored ^3^. Furthermore, although both donors and recipient parents are the initial consumers, the goal of creating a child who is clearly impacted by this market necessitates that we view the donor-conceived children as “consumers in trust”. Conceptualized as such, the stronger players, especially fertility clinics and parents, should be required and are intuitively entrusted with the responsibility to behave in ways that will protect the rights and interests of donor-conceived children so they can exercise them in the future.

The medical practice in the U.S. further gives rise to two other limitations that merit the regulation of this market. The first is the insufficient knowledge of genetics and genetic disorders among physicians. Studies consistently show that healthcare professionals, especially primary healthcare providers, including pediatricians and pediatric specialists, as well as fertility clinics are often lacking genomic literacy, and that they are unaware and untrained to make appropriate diagnoses of genetic diseases, especially rare ones [39,42–44]. In addition, when genetic testing is performed, healthcare professionals often express lack of confidence in interpreting the results and discussing genetic information with families [45]. The ability of front-line healthcare professionals to deliver health and genetic services to donor-conceived children is thus significantly curtailed.

The privatized healthcare system further exacerbates this problem. Although this system builds on referrals to specialists, who could arguably provide better genetic services, the reality is that the number of genetic counselors is insufficient to respond to the demand [45,46]. For instance, a survey of pediatricians in Alabama found that 24% were unable to obtain genetic consultations for their patients, 67% said that face-to-face genetics evaluation was difficult or impossible, and 58% reported that even a remote genetics evaluation would be difficult or impossible [42]. The privatized healthcare system in the U.S. further raises a challenge of affordability. Unlike other Western countries where genetic testing is subsidized through the national healthcare services, insurance companies in the U.S. may, and commonly refuse to cover the costs [39] ^4^. Further worse, this practical obstacle creates a conceptual barrier that is hard for patients to cross. Min-Jye Chen *et al.*, e.g., found that although pediatricians in the U.S. appreciate the value of genetic assessment, they may not order genetic testing or refer to a geneticist because of their concerns about the associated costs [47]. Thus, the donor-conceived child is the one to pay the price for this limitation: the time for a diagnosis and appropriate treatment are unnecessarily delayed. Clearly, the interests of these children are important.

Second, the lack of regulations rewards the wrong (and stronger) actors in this market, to the detriment of donor-conceived children. Because of the U.S. privatized and often unaffordable healthcare system, recipient parents may find a genetic diagnosis useful in so much as it would enable them to sue the clinics for negligence to recover the mounting health-related costs. This is not to suggest that fertility clinics are necessarily liable for a donor-conceived child born with a genetic disorder or to promote such a claim (see Section 4). Rather, it is to emphasize that the child's medical needs should supersede the financial interests of fertility clinics, and that the regulations of the latter would increase the diligence in which fertility clinics select gamete donors and are able to follow up with donors when such a medical diagnosis arises.

Indeed, in the lack of mandatory regulations, fertility clinics can neglect to follow up on medical information the donor provided, refuse to provide such information when requested by recipient families [48,49], or destroy the donors' file altogether. Furthermore, fertility clinics may and often continue to use the gametes also throughout the period in which symptomatic donor-conceived children are being under medical investigation. Once again, this practice is especially problematic in the U.S. as no limitation on the number of recipient families that can receive donation from the same donor is set. Thus, for instance, in one reported case, the sperm of a donor with a genetic heart disease was used with at least thirteen families and produced twenty-two donor-conceived children, eight of which were diagnosed with the same genetic disorder (two died) [50]. In another reported case, gametes from one single donor was used to conceive forty-three children, five of which inherited the donor's neurological disorder [44]. Put differently, the primary beneficiaries of the regulation-free market in the U.S. are gamete donors and fertility clinics who are in the business of making money, rather than the donor-conceived children who are—or are at risk for being in the future—in medical need. Thus, regulating this market is justified to prevent donor-conceived children who experience health symptoms from falling through the cracks and to prevent further use of gametes diagnosed with genetic disorders.

## 3. The “Childs's Right to Know”: Challenges in the U.S

Another emerging justification for the regulation of the market for assisted reproductive technologies in the U.S. is the suggestion that donor-conceived children have (or should have) a right to know about their genetic origin. Proponents of disclosure such as Lucy Frith, Vardit Ravitski, and Naomi Cahn, suggest that this “right to know” should be recognized as an inherent and universal right, including a right to know about one's circumstances of conception, and a right to know information including identifying information about the donor [2,4,6]. Subsequently, although most countries in the world still uphold the policy of donor anonymity, there has been a visible international trend to reverse it. European, especially Scandinavian, countries as well as New Zealand and Victoria, Australia, have taken the lead, enacting laws that require gamete donors to provide both their medical and identifying information and to a-priori consent to be contacted by any resulting donor-conceived child in the future [9–13,51]. Victoria, Australia, further requires that an addendum is attached to the birth certificate of donor-conceived children, informing them about the donation and the availability of information about the donor [13,52]. A similar proposition in the UK failed [53].

In the U.S., recognition of such a right to know raises legal and ethical difficulties. Among the hallmarks of American society are the values of (adults') reproductive freedom (in this case, of the recipient parents) and privacy rights of both the donors and the recipient parents. Moreover, the concept of familial privacy is strongly endorsed. Parents generally have a broad prerogative as to how to raise their children and to make decisions on their behalf, including in critical issues such as medical treatment. Although these concepts exist elsewhere, they are entrenched in the American psyche of individualism and self-proficiency—commonly contrasted to the European philosophy of state welfare/socialism—and have significant implications for children [54]. Indeed, the U.S. legal system makes only little room for children's rights. Unlike other Western counterparts that explicitly endorse children's rights in their supreme legal framework [55,56], the U.S. neither ratified the United Nations Convention on the Rights of the Child [57] nor includes any mention of children as subjects of rights in its Constitution. Children's rights are mostly based on judicial interpretive processes, which evolved primarily in areas where there are strong societal interests rather than (or, not exclusively) because minors' interests (e.g., abortion) [56,58]. Conversely, it is well-accepted that parental prerogative is not unlimited, and courts generally support intervening on parental rights when the child's physical health or safety is in serious danger [59]. The question is thus whether in the context of donor-conceived children such serious welfare-related harms exist and justify overriding parental prerogative.

In this regard, disclosure proponents commonly raise three types of potential harm to the donor-conceived child, in addition to the medical harm already discussed. The first is familial harm, which focuses on the importance of the value of truthfulness. It suggests that family secrets create ongoing tension, taint the familial communication, and negatively impact the family relations [60]. The second is identity harm. As Vardit Ravitski, e.g., suggests, one's genetic information is tied with one's identity formation [4]. Analogizing gamete donation to adoption, this harm argument suggests that donor-conceived children can experience poor self-perception and identity crisis [6,61]; some have suggested that this amounts to so-called “genealogical bewilderment” [33]. Finally, the third focuses on kinship. It suggests that genetics intrinsically makes U.S. feel emotionally close to our kin, and that to prevent “genetic alienation”, a donor-conceived child has a right to materialize this intrinsic connection through the provision of information and especially the ability to develop a relationship with the genetic parent [4,7].

The remainder of this article considers whether these harms justify overriding parental prerogative. The emphasis is on the U.S. context (rather than general or abstract consideration), focusing on three issues. The first concerns the weight granted to genetics. It is, of course, undeniable that genetics impacts issues such as appearance, temperament and propensity to develop certain health conditions. However, the argument, as advanced by disclosure proponents, goes deeper. It suggests that genetics is a prime—even *the* prime—factor that determines who one is, and thus justifies trumping the interests of others. Indeed, a right to know and the subsequent harm of not knowing emerge only if genetics is upheld as indispensible, and a prerequisite, to one's personhood. Such a genetic-essentialist understanding of one's self and identity is misleading, however. It overstates individual's genetic make up and insinuates that one's “authentic” identity and personality traits are grounded merely in one's genes—although scientific knowledge has proven this wrong [62]. It also downplays other factors that are critical in the development of identity, especially the role of society and the environment. Indeed, perhaps the biggest message of literature on disability, genetics, and identity is how multifaceted the process of identity formation is, and how societal exclusion is influential in this process [63].

Further significant is how culturally constructed is the notion of the genetic tie and its importance more generally. One example ^5^ is the case of egg donation in which the recipient mother carries the pregnancy. In such scenarios, both the genetic and gestational mothers have a biological role in the resulting child. However, while emerging studies in epigenetics show that the gestational environment influences how the fetus's genes are later expressed [64], Susan Golombok et al's study of children born through reproductive technologies found that, at age 7, surrogacy children show higher levels of adjustment difficulties than children resulting from egg or sperm donation [65]. Determining whether, for the child, it is really the genetic rather than gestational tie that is critical, or whose maternal contribution “counts more” for the child's identity depend on one's construction of motherhood and is thus impossible. Another example is Orthodox Judaism construction of fatherhood. Under this religious stream, non-Jewish men who have a (genetic) child with a Jewish woman are not held as having any paternal relatedness with the child [66]. There is subsequently a denial of the need to know, and from the perspective of members belonging to such communities, any arguable harm by not knowing is odd.

This is not to say that the position of parents and other figures belonging to such religious communities should necessarily prevail. For donor-conceived children who are raised in such religious communities and who adopt a similar worldview, non-disclosure of one's genetics origins may be the appropriate decision. However, the views of recipient parents and donor-conceived children (especially mature ones) might not be in sync. Because children are separate human beings from their parents, it is impossible to unquestionably assume that they will follow the parent(s)' religion and ideology. Children may opt out of the religious community and, as studies show, may develop their own identity, separate from their parents [67]. Regardless of parental preferences and religious ideology, then, donor-conceived children may indeed be curious about their genetic origins. In fact, the call for a child's right to know has emerged seemingly because of such instances: when disclosure of the child's circumstances of conception occurred in traumatic circumstances, such as during parental divorce proceedings or from a third party, and the donor-conceived child subsequently developed an identity crisis akin to that documented for adopted children [68]. For such instances, an organized registry of gamete donors would enable the child to access information about the donor—even if not necessarily identifying information—if she finds it fit. Put differently, further regulations of gamete donation in the U.S. can provide the framework to protect the interests of donor-conceived children (and future adults) without classifying these as a right to know, which current American legal system and culture are likely to resist.

A second issue to consider is the scope of the right to know and what such a right actually means. Obviously, one's circumstances of conception are critical for the child's right to know. Yet if knowing this includes a detailed description–from gamete donation, to parental use of medication, in-vitro fertilization (IVF) treatment ^6^, and so forth—it would altogether eschew parental privacy rights. It is further doubtful whether knowing about one's circumstances of conception would be enough. Testimonies of some donor-conceived children suggest that knowing about their circumstances of conception but having no access to information about the donor produced anxiety and self-doubts about who they are [61,68]. While meeting with half-siblings through on-line listserves (such as the Donor Sibling Registry [69]) may mediate some of the frustration, the relief is only partial, suggesting that more information is needed. Yet the concept of a right to know does not clarify what else, besides circumstances of conception, one has a right to know.

Surely, it would include the donor's medical history as collected by the fertility clinic that provided the service (see discussion above). But as the study of the human genome develops, one may equally argue that the medical file should include donor's genomic data (including, e.g., information about behavioral traits), or that the donor be tested for genetic disorders whenever appropriate technologies develop to include this information in the medical file. However, genetics information is often unknown to the donor him/herself—even if only out of fear from discrimination [39]. Although the Genetic Information Nondiscrimination Act of 2008 (GINA) [70] unified the ban of genetic-based discrimination in employment and health insurance across U.S. states, the Act has some important limitations [71]. Moreover, studies suggest that public fear from genetic-based discrimination is high [72,73]. Recent recommendations from the American College of Medical Genetics and Genomics about clinicians' duty to report incidental findings of genetic mutations [74] further complicate the issue. While the ACMG has listed fifty-seven genetic mutations that are clinically actionable (*i.e*., when medical intervention exists), donors may practically be forced to receive information they may not want to know. In effect, then, the privacy rights of donors, and their right *not* to know, would be subjected to the child's right to know.

Even if access to a donor's genetic information may be justified when donor-conceived children experience various medical symptoms, the implications of providing such information, which may go beyond the recommended list of the ACMG, also to donor-conceived children who are asymptomatic, merits caution. As Klitzman's extensive study of adults who underwent genetic testing reveals, whether knowing about one's genetic composition is helpful and desirable depends on a myriad of factors, including personality, family and social circumstances, the availability of treatment, and others [39]. How such information will be disclosed to asymptomatic donor-conceived children is a further reason for concern. Given online listserves for recipient parents and sibling donor-conceived children, it is crucial to ensure that information about potential genetic disorders is not merely posted on the web but provided by experts who can explain the implication thereof and provide the needed support.

This challenge is more pronounced in the U.S. given, on the one hand, the privatized healthcare system with its limitations, as described above, and on the other hand, the burgeoning market of direct-to-consumer genetic testing companies. In particular, studies suggest that genetic testing of children through the latter is increasingly viewed as a parental prerogative [75], and that some of the leading companies (e.g., 23andMe) in fact also target young consumers (ages > 13) when designing and marketing their genetic services [76]. These companies further increasingly offer an array of social networking opportunities and encourage consumers to share their genetic data through these platforms [71]. Notwithstanding the FDA's 2013 suspension of marketing of health-related genetic testing by 23andMe on the ground of lack of clearance for scientific accuracy of testing [77], it recently granted the company the first permit to provide a genetic carrier test for Bloom syndrome [35], and the expectation is that additional permits will be granted over time as technologies are more accurate. However, while these direct-to-consumer companies will be able to provide (especially in the future, as the tests are also offered at lower costs) an alternative to families who are uninsured or whose insurance companies denied the request for testing, these companies do not currently provide professional support to understand the genetic implications to consumers. The concern that donor-conceived children (especially adolescents) will use these private services to connect with genetic kin but end up learning unexpectedly about their proclivity for certain genetic conditions without being provided with medical and other supports in this disclosure is thus heightened.

Moreover, this possibility raises a concern that the child's right *not* to know may be unintentionally violated. The right not to know, which is based on notions of individual autonomy and privacy, is well established in Western medical practice, especially in the context of genetic data [78]. However, given that these direct-to-consumer companies do not have a mechanism in place to ensure that consumers understand the implications of their consent [71], it is unclear, indeed, doubtful, that donor-conceived children who access these online services are well informed about the possibility that in searching for genetic kinship they may forfeit their right not to know about genetic proclivity for disorders. Once again, then, regulations of this market, including FDA oversight of direct-to-consumers genetic services and mandatory counseling, could alleviate the concern and protect the legitimate interests of donor-conceived children without classifying this as a child's *right* to know that is unlikely to be endorsed in contemporary American legal culture. Such regulation will also be in line with U.S. requirement of informed consent and rationales for regulating economic markets (indeed, it is for these rationales that the ability of such direct-to-consumer companies to provide health-based services is currently significantly limited [79]), and thus easier for the American public to adopt.

The conceptualization of access to donor's medical and genetic information as a right is further problematic as it suggests that naturally-conceived children should also have a right to access their parents' medical information [80]. After all, by definition, a right is an entitlement that all children should equally have. Yet no such right currently exists ^7^. Furthermore, given that medical issues, especially genetic conditions, are often not shared also with other members of the family [39], acknowledging such a right would create a revolution with regard to privacy in medicine which would be very difficult to implement. The challenge is, therefore, if and how to support donor-conceived children without simply replacing the privacy rights of both recipient parents and donors with however we conceptualize the right to know, and indeed, without unintentionally harming donor-conceived children by providing them information they might *not* want to know.

A third dilemma with the right to know concerns the procedure for disclosure. If the child has a right to know, there should be a mechanism in place to facilitate the child's knowledge of her/his circumstances of conception and access to donor information. As a society, there also needs to be a decision as to who has the duty to disclose the circumstances of conception and other data about the donor to the child. Of course, truthfulness is often regarded as an important virtue. It is a core principle in Western bioethics, where it is part of the doctrine of informed consent, and, as mentioned, children's advocates suggest that it is paramount for healthy family-relations. There is therefore an expectation that parents will do “the right thing” for their child and disclose the story of conception.

Most laws that regulated gamete donation and follow this idea (e.g., the Netherlands [10], the UK [11]), have adopted so-called “passive registries” [80]. Although these laws require that providers of reproductive services record some identifying information about the donor, the donor-conceived child is the one who is expected to initiate the release of information at maturity (usually assumed to be achieved at 16–18 years old). The problem with such registries is that their usefulness is typically limited to nontraditional families (single parent or same-sex couples), where the use of gamete donation is naturally more obvious, or to other instances where the donor-conceived children already know and can initiate such disclosure proceedings. However, they are significantly less useful with heterosexual couples who resort to gamete donation. Indeed, studies show that the preponderance of donor-conceived children born to heterosexual couples do not know about their genetic origins [68]. Thus, if a donor-conceived child indeed has a right to know, there should be a system in place to ensure that the child is informed about his/her genetic origins.

One such option is to obtain the prospective recipient parents' commitment to disclosing the gamete donation to the child. As Adrianne Asch suggested, only once this condition is satisfied, parents will be “licensed” to proceed with the procedure and use donated gametes [8]. Although this may provide an initial solution, it is limited in that it does not include an enforcement mechanism. Studies in the UK, for instance, found that although recipient parents are encouraged to disclose the gamete donation to the child, only 30%–40% of heterosexual couples intend to do so [81,82]. Further, heterosexual recipient couples that pre-procedure express an intention to disclose the gamete donation to their child may not follow suit. Thus, an additional implementation procedure of the child's right to know will be needed, that is, it will require that an “active registry” be adopted [80]. Under such a registry, gamete banks or another body would contact the donor-conceived child at the age of maturity. Another option would be an addendum to the child's birth certificate, stipulating his/her circumstances of conception, as adopted in law in Victoria, Australia, and debated in the UK [13,52,53]. This would ensure that donor-conceived children indeed know and can request the donor's information later on.

However, the extent to which these regulations can and should be adopted in the U.S. requires consideration of three related issues. First, an active registry inherently assumes, as commonly also implied in the scholarly work, that truth-telling in the context of gamete donation and the subsequent policy of disclosure is an absolute good. In practice, however, truth-telling—including in the realm of medical ethics—is relative. For instance, as Ruth Macklin points out in her work on relativism in medical contexts, physicians may avoid saying the word “cancer” to a patient who is terrified of dying from the disease when she is diagnosed with a benign tumor, if full disclosure would lead to a crisis [83]. Similarly, there is no consensus whether physicians who during medical diagnosis and treatment of child incidentally discover that there is no biological relationship between the parent (usually, father) and child (so-called: “misattributed paternity”) ought to disclose this information (and to whom) [80,84]. In some cases, the healthcare providers decided to disclose this information to the family members involved; but in others, the medical team found it to be irrelevant [85–87].

Comparable “relativist” justifications may exist in the context of gamete donation. Among Orthodox Jewish and Muslim communities, for instance, the pressure to procreate may be so high that a husband may be religiously or culturally obliged to divorce his wife on the basis of infertility [20,88]. Concurrently, the use of gamete donation may be viewed as prohibited adultery with significant ramifications on the social and religious status of both recipient parents and the donor-conceived child, who may then be ostracized from the community altogether [20,55,66,88,89]. While discussing the option of disclosure with such recipient parents who nonetheless decide to procreate by using gamete donation would be appropriate, insisting on truth-telling, especially when there is no medical necessity, may have unnecessarily high costs for both recipient parents and the child. Such insistence will also contradict the U.S. approach, which prides itself as being a “melting pot society” and requires respect of religious and cultural diversity in private lives.

Such interference in parental disclosure decisions is especially troubling, given that as a practical matter, it is questionable whether not knowing indeed harms the child at all. As mentioned, there are *some* testimonies of donor-conceived children who expressed frustration and identity doubts because, although they know about their conception story, they do not have access to information about the donor. The frustration and agony of such children should be taken seriously by all involved in the process, and the regulations of gamete donation as suggested may facilitate greater openness in the future. However, this is by no means universal. Studies with children born as a result of sperm donation, and raised by families with various structures show mixed results. Interestingly, diversity in views and preferences exists also among donor-conceived children who are raised together in one family [90]. Some prefer anonymity; others are satisfied with non-identifying information; and yet still others want to know identifying information about the donors [90–92]. Moreover, to my knowledge, there are no studies of the relative occurrence of identity crisis among the general population of donor-conceived children, including especially the millions who do not know about their genetic origin, nor about whether their identity crises are any different than what other children experience as they go through their adolescence years. On the contrary: as Golombok has found in her decades long research of children conceived by sperm and egg donations, including some who do not know about their conception story, the quality of family relationships and children's socio-emotional adjustment and psychological development to be within the normal range [93–95]. The researchers subsequently concluded that these family relationships and subsequent child development “do not represent a cause for concern” [94]. Thus, there is a concern that by prioritizing truth-telling as an absolute good, we are sanctioning an intrusion into family relations and children who are otherwise flourishing.

A second issue regarding disclosure and active registries concerns the role that physicians should have in upholding the child's right to know. That is, the conceptualization of data about one's genetic origins and disclosure of donors' data *as a* right suggests that there is—or should be—a parallel societal obligation, including physicians' duty, to uphold the child's right to their best of their abilities. Indeed, in countries that adopted a gamete donation disclosure policy and active registry, there is a national/state level registry, and physicians are required to notify regarding the patients and successful births [9–13,51]. Such notification by physicians is possible on a practical level also because fertility services, including gamete donation, are provided as part of the national healthcare system. Neither of these conditions exists in the U.S., however. Physicians in the U.S. are not normally required to disclose identifying information about patients to third parties, including under the Center for Disease Control's National ART Surveillance System [96] ^8^. While exceptions exist (for instance, reporting on lethal infectious diseases [97] or when a patient may be harmful to others [98]), it will be hard to expand it to individuals and couples who are going through a fertility treatment, even though they do not have medical conditions which are risky to others and regardless of their consent. Indeed, such physician's notification will require revamping confidentiality laws in medicine as currently exist in the U.S.

Such surveillance is also significantly more complicated in the U.S. because of the fragmentation of the healthcare system. Given the privatized healthcare system, infertility services and treatment during pregnancy are commonly carried out by different physicians, and often, in different states. One possible way to overcome this fragmentation is to require that all newborns be tested to ensure that they have genetic compatibility with their presumed parents, and that the results of these tests will be recorded on the birth certificate. This option is clearly highly intrusive in family life, and given the estimates that ten percent of children born in the U.S. to heterosexual couples are unknowingly raised by non-biological fathers [99], it is also unlikely to pass American standards of privacy.

Finally, the third consideration regarding the disclosure of donor's identifying information and active registry concerns the impact it will have on the overall market. Given that many gamete donors (especially sperm donors) are uninterested in the resulting child [68] ^9^, and that they often donate mostly for financial reasons [100], all countries that reversed the policy of anonymity encountered two challenges: a significant drop and subsequent shortage in gamete donation, especially for ethnic minority groups [29,101,102], and simultaneously, an increase in fertility tourism where those who can afford it travel to the U.S. and to other destinations which are less regulated and where donors' anonymity is upheld [28]. Also, the demographic characteristics of gamete donors have changed. Studies suggest that sperm donors tend to be older and often married, with some also reporting higher rates of men with mild psychopathology and alcohol abuse [68]. Such characteristics are clearly less favorable for a donor-conceived child. Thus, regulations of gamete donation in the U.S. should take into account how to balance the various interests of donors, recipient parents and donor-conceived children, and, possibly, also the market as a whole.

Revisiting the process of donors and recipient-parents' informed consent may facilitate this goal. First, this process could include encouragement of gamete donors to leave some personal information in their file at the time of donation (similar to adoption [2]), and service providers may further create a mechanism that allows donors to provide such information later on. In this way, donors who initially opted for anonymous donation but changed their minds over time (which may happen, especially as donor-conceived families become even more accepted) will be able to revisit their decision. Second, the type of information provided to stakeholders should be reconsidered. In addition to the conventional requirements of data about the medical procedure, risk, and benefits, service providers may include research about donor-conceived children and references to other sources (e.g., the Donor Sibling Registry's website) that would better inform these stakeholders about the needs and interests of (some) of the resulting children. An opportunity to meet or talk with recipient families who can share their experiences could also be offered. Although not currently offered elsewhere (to my knowledge), such a mechanism to promote informed decisions exists in other medical contexts. For instance, the Federal government and a few states in the U.S. have enacted laws requiring that prospective parents who undergo prenatal genetic testing and find that their fetus has Down Syndrome are offered to meet with families with and organizations providing services to such children before deciding whether to abort or continue the pregnancy [103–105]. Although these laws raised some concerns (predominantly because they are viewed as a disguise for pro-life/anti-abortion philosophy) [106], the value of increased information is uncontested [107]. A similar opportunity could thus be extended to gamete donors and recipient-parents. Given that some recipient parents may desire more information about the donor (either for themselves or because of their child's quest) [17] or be unaware of the possible ramifications of lack of such information, such a pro-information mechanism may further empower them as consumers to demand a more transparent process, reduce the costs of purchasing gametes from non-anonymous donors (which are currently higher than gametes from anonymous donors [2]), and mobilize the market in a different direction.

## 4. Broader Social Implications

In addition to the challenges to medical ethics that arise in light of the child's right to know, the recognition of such a right might also have broader social implications which merit consideration.

The first is that a right to access donor's medical and genetic information may give the wrong impression—indeed, illusion!—to recipient parents (and to society more generally) that they are guaranteed to have disability-free children. A language of rights suggests that there is a corresponding duty on parents and, by extension, on gamete donors and fertility clinics, to know about and to avoid passing along genetic disorders. Certainly, this is not a new debate [80,108–111]. It arose with the genetic revolution and gave rise to the possibility of so-called “wrongfully disabled” cases, where children sue their parents for being born with a genetic disorder that parents could have theoretically prevented through the use of technology. Courts thus far have refrained from accepting such cases [48,112]. However, if access to parental medical and genetic information is re-conceptualized as a right, it may spur new interest in such “wrongfully disabled” suits. This will require judicial resolution of questions about whether there is or should be a parental duty to prevent harmful conditions in their child, including a predisposition to genetic disorder; what sort of disabilities give rise to such a parental duty; and by extension, what are the reasonable expectations from gamete donors and fertility clinics in testing for genetic disorders. However, given that a “positive genetic test” rarely provides information about the actual likelihood for the disorder to occur, its severity, and the expected time for onset of the associated disorder [34,113], the answers to these questions are not obvious.

Further, given that there is no study showing that the rate of donor-conceived children with disabilities is higher than what it is in the general population and that disability is a natural phenomenon, the implied message about disability-free children as encapsulated in the right to know is concerning. As many persons with disabilities and their advocates have pointed out, such claims send the (wrong) message of lesser value in a life with disabilities [8,114,115]. It may further exacerbate societal stigma and discriminatory attitudes towards persons with disabilities. Indeed, while there is strong and consistent evidence that the “geneticization” of conditions such as psychiatric disorders increases stigma [116,117], it is often also the case that the genetic identification of such complex disorders is impossible at this point to achieve.

Second, the implications of the right to know on the societal construction of the family should be considered. Theoretically, recognizing the right to know reflects well upon the reality of the new families. It acknowledges the donor's contribution, which enabled the creation of these families, regardless of their exact structure. It is also tuned to studies showing that children's sense of familial attachment often adjusts to their familial structure and that, for children, the most important thing is that they were planned and wanted all along [118–120]—qualities which are undisputable in the context of assisted reproductive technologies, gamete donation included. Thus, a right to know also sends an important message of acceptance of familial diversity in society, and should be promoted.

In the U.S., however, a right to know may serve as a double-edged sword vis-à-vis the legal and societal constructions of the family. Notwithstanding the recent landmark decision of the U.S. Supreme Court that legalized same-sex marriage across the states [121], social acceptance of family diversity is not automatic. In this regard, and even though it may not be intentional, the emphasis on one's genetic origins may reinforce the traditional family comprised of a genetic father and a genetic mother as the ultimate family structure and undermine other non-traditional family structures of these donor-conceived children [122]. Conversely, a right to know intuitively moves towards a reality of a “multi-parents” family model [123,124]. Even if gamete donors do not currently have a say in the child's rearing, a donor-conceived child who matures might choose to have some sort of a parental or other relationship with the donor, and donors might find that a relationship with their genetically-related children is nonetheless desirable [125]. However, this intuition immediately encounters the “two-parents-only model” which is prevalent—and paramount—in family law and culture in the U.S., which limits the number of the legally recognized parents—whether heterosexual or same-sex parents—to two [126–129]. While a legal overhaul of the two-parents model may not be needed, and in most likelihood, also undesirable to many donors and recipient parents, encouraging openness about alternative or more fluid parenthood structures is appropriate. Indeed, studies of the increased trend in adoption and surrogacy agreements to preserve some contact between the adoptive/surrogacy families (both parents and children) and the genetic and gestational parent(s) indicate that even little contact improves the overall positive experience of all involved [130,131]. Conversely, by recognizing a right to know without making appropriate adjustments in family laws and without creating the societal acceptance of such families, the dissonance between law, society and medicine in the construction of the family may be exacerbated. Once again, the prime victims are the resulting children.

## 5. Conclusions

There is no question that scientific developments in reproduction have changed the landscape of the family. Gamete donation is no exception, and it requires that attention be given to the medical, familial and social implications thereof, including the resulting children.

Medical needs of donor-conceived children, coupled with general rationales for regulating economic activities and more specific deficits in medical practice in the U.S., provide strong justifications for the regulation of this market. This is especially true given the privatized and fragmented healthcare system in this country. Simply stated, the existing system considers the interests of all but the donor-conceived children, who are the only ones who have no say as to whether to be born. Regulations of this market may also enable addressing other challenges, and balancing the interests of stakeholders, that are unique to the U.S. system. These include respect for multicultural societies while upholding donor-conceived children who may develop religious and cultural perspectives that are different than those of their parents', protecting their interests as “consumers in trust”, and safeguarding them given the rise of direct-to-consumer genetic testing market and its tempting but possibly harmful social networking options.

A national, comprehensive, legislation relating to gamete donation—akin to that in other developed countries—is likely unfeasible in the U.S. at this this point. However, there is room for increased regulations, which would be in sync with the U.S. creeds. Without intending to be exhaustive, some suggestions to this affect include increasing genetic and genomic literacy among physicians, especially Ob-Gyn, pediatricians, and service providers in general. More oversight by Federal agencies, including the FDA and the Center for Disease Control about the type and extent of donors' data that is collected (and preserved) and the possibility of follow up with donors and later update of donors' files. In this way, regardless of whether recipient parents and donor-conceived children are aligned in their quest for donors' information, some recourse for donor-conceived children who want access to donors' information will exist. Additionally, recent studies found that non-anonymous gamete donors express a need for counseling also a few years post-donation [132], and ensuring access to such services will be important. However, as suggested earlier, it is also critical that mandatory genetic counseling is provided to recipient parents and mature donor-conceived children, especially those who are using online services and direct-to-consumers companies in search for genetic kinship. This will protect donor-conceived children from unknowingly forfeiting their right not to know about genetic conditions that may be traced back to the donor. Given the U.S. legal, political, and free-market market cultures, a fully centralized registry of gamete donors will be difficult to achieve. It may also lead to a major drop in supply—a backlash that powerful actors in the U.S. reproductive market, including prospective recipient parents, will certainly resist. However, pro-information laws and mechanisms to improve stakeholders' informed consent process are entirely within the U.S. rationales for regulating markets and principles of medical ethics. Raising awareness about the possible needs of the resulting children could also empower prospective parents/consumers to demand greater transparency and reduce antagonism among donors.

Whether there is a moral-translated-into-legal donor-conceived child's right to know requires further consideration, and it is particularly complicated in the U.S. given its culture and legal framework pertaining to children's rights. The moral backdrop of such a right is complicated, given existing religious and cultural pluralism in the U.S., and the legal and societal value of individualism, reproductive freedom, non-interference in family matters, and (adults') privacy rights that in general, supersede children's rights. A legal right is further fraught with an array of medical, practical, and social dilemmas as to what it actually means, how it can be implemented, and at what benefit—and cost. Whether truthfulness is an absolute good, how to balance between access to donor's medical and genetic information and privacy in medicine of parents and donors, and what role physicians have—or should have—in upholding this emerging right to know all suggest that rethinking some fundamental principles of contemporary medical ethics in the U.S. is needed. The broader social implications of this right to know, especially the implied guarantee of disability-free children, the implications of geneticization on stigma and discrimination against persons with disabilities, and the societal construction of the family (along with the appropriate legal measures to support it) are too important not to consider.

There is much room for further research is this field, not least by adding the perspectives of donor-conceived children across family structures and religious/cultural diversity including the voices of donor-conceived children who do not know their conception story. Fleshing out questions concerning disclosure of genetic information and diagnosis to donors and donor-conceived children should further be explored, along with the responsibility of fertility clinics in doing so. The debate surrounding disclosure of gamete donation raises too many interests, concerns, and duties to ignore.

## Footnotes

1 In the U.S., the payment peaks at $50,000 for egg donors and at $2,950 for sperm donor (per cycle) [28]. For comparison, in the UK, Israel, and France, for instance, gamete donation has to be altruistic; financial compensation of gamete donors is limited to expenses and inconveniences directly associated with the donation.

2 Dr. George Annas coined the notion of Wild West in medicine, which has been subsequently extensively used by others in an array of areas, including reproductive technologies (see [30]). Here, the notion of Wild Wild West followed Rosas's title [19].

3 I thank anonymous reviewer 2 for highlighting this point.

4 The Patient Protection and Affordable Care Act of 2010 (known as “Obamacare”) does not change the previous decision-making process of insurance companies. Whether these insurance companies approve or reject the need for genetic testing depends on their judgment (correct or not) as to whether the procedure is medically required. Patient Protection and Affordable Care Act, Pub. L. No. 111–148, 124 Stat. 119 (2010) (codified as amended in scattered sections of 21, 25, 26, 29, and 42 U.S.C.).

5 I thank anonymous reviewer 1 for a comment that instigated this example.

6 In *in-vitro fertilization* the woman's egg is fertilised by sperm outside of the body, in a Petri dish, and then implanted in the womb.

7 See however Safer v. Estate of Pack (I), 291 N.J. Super. 619, 677 A.2d 1188 (App.Div.), certif. denied, 146 N.J. 568, 683 A.2d 1163 (1996).

8 Fertility clinics are required to provide information on pregnancy outcome to the Center for Disease Control as part of the National ART Surveillance System; however, patients' information such as name and address is not reported and remains confidential.

9 Women have expressed greater interest in future offspring.
